# Multi-Sensor Fusion Framework for Reliable Localization and Trajectory Tracking of Mobile Robot by Integrating UWB, Odometry, and AHRS

**DOI:** 10.3390/biomimetics10070478

**Published:** 2025-07-21

**Authors:** Quoc-Khai Tran, Young-Jae Ryoo

**Affiliations:** 1 Faculty of Electrical and Electronics Engineering, Vietnam Aviation Academy, Ho Chi Minh City 70000, Vietnam; khaitq@vaa.edu.vn; 2 Department of Electrical Engineering, Mokpo National University, Jeonnam 58554, Republic of Korea

**Keywords:** indoor localization, sensor fusion, Ultra-Wideband (UWB), wheel odometry, Attitude and Heading Reference System (AHRS), Kalman Filter, trajectory tracking, mobile robot, Dynamic Time Warping (DTW), real-time navigation

## Abstract

This paper presents a multi-sensor fusion framework for the accurate indoor localization and trajectory tracking of a differential-drive mobile robot. The proposed system integrates Ultra-Wideband (UWB) trilateration, wheel odometry, and Attitude and Heading Reference System (AHRS) data using a Kalman filter. This fusion approach reduces the impact of noisy and inaccurate UWB measurements while correcting odometry drift. The system combines raw UWB distance measurements with wheel encoder readings and heading information from an AHRS to improve robustness and positioning accuracy. Experimental validation was conducted through repeated closed-loop trajectory trials. The results demonstrate that the proposed method significantly outperforms UWB-only localization, yielding reduced noise, enhanced consistency, and lower Dynamic Time Warping (DTW) distances across repetitions. The findings confirm the system’s effectiveness and suitability for real-time mobile robot navigation in indoor environments.

## 1. Introduction

Mobile robots primarily operate in indoor environments where reliable localization is essential for autonomous navigation. Recently, biomimetics-inspired navigation technologies have gained significant attention, leveraging biological principles to enable robots to perform intelligent localization and positioning—much like animals navigating toward food, shelter, or safety. The operation of a mobile robot—exploring unknown environments, identifying targets, and executing tasks—closely parallels the adaptive behaviors observed in biological organisms.

Animals demonstrate remarkable robustness in navigation through a combination of decision-making, action selection, spatial representation, perception, and exploration. These capabilities allow them to perform complex tasks such as foraging, homing, and hunting in dynamic and uncertain environments. By mimicking these biological strategies, robots can enhance their autonomy, adaptability, and resilience in indoor settings.

This study focuses on localization and positioning techniques for mobile robots. Accurate localization is one of the most critical capabilities in automated mobile robots, as it underpins core functionalities such as motion control, path planning, and navigation in structured and unstructured environments. In indoor scenarios where GPS signals are not available, a variety of positioning technologies have been explored, which can broadly be categorized into three approaches: proximity-based methods, scene analysis, and anchor-tag systems.

Proximity-based systems, such as magnetic spots, QR codes, and RFID tags, allow robots to estimate their position when near known reference points. Although these systems are relatively simple to deploy, they are limited in scalability and flexibility, especially in large or dynamic environments [1,2]. Scene analysis methods, including Simultaneous Localization and Mapping (SLAM), typically rely on LiDAR or visual sensors to build environmental maps and simultaneously localize the robot [3]. While SLAM provides high localization precision, it is computationally intensive and consumes significant power, which is undesirable for lightweight and energy-constrained platforms. Anchor-tag systems estimate position by measuring distances between a mobile tag and fixed anchors using techniques such as laser scanning or radio signals. Laser-based localization systems offer high accuracy but pose health and safety concerns due to laser exposure in human-inhabited environments. In contrast, radio-based systems—particularly those using Ultra-Wideband (UWB)—are considered safer and have gained increasing attention. UWB is a short-range radio communication technique operating over a wide frequency spectrum, capable of delivering centimeter-level accuracy using Time of Flight (ToF) ranging methods [4].

Despite the advantages of Ultra-Wideband (UWB) systems in delivering centimeter-level localization accuracy, their performance significantly degrades under non-line-of-sight (NLOS) conditions, multipath propagation, and electromagnetic interference, reducing overall reliability and robustness [3,4]. To overcome these challenges, recent studies have adopted multi-sensor fusion approaches, combining UWB with wheel odometry, inertial sensors (IMU), ultrasonic sensors, or even vision-based inputs [5,6].

A wide range of fusion strategies has been proposed. Shalihan et al. [5] proposed a robust indoor localization system that fuses UWB with LiDAR-based laser odometry and wheel encoders through pose graph optimization. Although effective in dynamic environments, this approach relies heavily on computationally intensive graph-based optimization techniques and requires high-quality LiDAR data, which may be cost-prohibitive or unsuitable for compact mobile platforms. Similarly, Zhang et al. [6] employed a factor graph-based method to integrate UWB, IMU, and ultrasonic sensors, achieving a 38% improvement in localization precision. However, their approach demands complex modeling and real-time factor graph updates, limiting its scalability and implementation on resource-constrained systems. In aerial robotics, Hadjiloizou et al. [7] demonstrated a multi-sensor fusion framework that combines UWB, IMU, and vision-based inputs to support the localization of quadrotors in environments with intermittent communication. While the integration of visual data improves pose estimation accuracy, it also introduces dependencies on sufficient lighting conditions and increased power consumption, making it less ideal for ground-based mobile robots operating in low-light or occluded environments.

Other approaches using Kalman filters for UWB–IMU fusion face their own limitations. For example, Liu et al. [8] investigated UWB-IMU fusion using a Kalman filter with velocity-based outlier detection; however, the lack of explicit heading correction in their system limits its robustness during long-duration navigation tasks. Similarly, Zhu et al. [9] proposed an AGV positioning method where the heading angle is indirectly estimated by geometrically calculating the relative positions of two UWB tags mounted on the robot. To improve the accuracy and robustness of heading estimation, both the wheel odometry and the UWB-based geometric heading were fused within a tightly coupled iterated Kalman filter (TEKF) framework. Although this fusion mitigates some short-term fluctuations, wheel odometry remains prone to drift over time due to integration errors and wheel slippage. Furthermore, the method’s reliance on UWB measurements exposes it to potential errors caused by signal degradation and calibration inaccuracies. More recent techniques, such as nonlinear stochastic filters [10], constant velocity models [11], and dynamic feasible region estimation [12], have been proposed to improve UWB–IMU fusion. However, many of these approaches remain simulation-only or lack critical components such as heading feedback from AHRS, which undermines their long-term robustness. Bao et al. [13], for instance, introduced a 3D localization system combining UWB and dual barometric altimeters. Although their method achieved promising results in controlled environments, it was not tested on a mobile robotic platform and did not include dynamic motion validation or integrated control.

Taken together, these studies highlight that using UWB and IMU alone is insufficient for stable and reliable localization under real-world indoor conditions. For ground robots, integrating UWB with wheel odometry and Attitude and Heading Reference System (AHRS) offers a more practical and scalable solution. Wheel odometry provides high-rate incremental motion estimates but accumulates drift, while AHRS—fusing data from accelerometers, gyroscopes, and magnetometers—offers robust heading correction to mitigate odometric errors [14,15,16].

Despite recent advancements in localization technologies, limited research has focused on the simultaneous integration of UWB, wheel odometry, and AHRS sensors to fully exploit their complementary characteristics. To bridge this gap, this paper presents a unified UWB-odometry-AHRS fusion-based localization system. In contrast to more complex approaches such as factor graph optimization or SLAM, the proposed system utilizes a standard Kalman filter for real-time sensor fusion, thereby simplifying the overall implementation. This approach harnesses the strengths of each sensor modality: UWB provides absolute position updates, wheel odometry offers reliable short-term motion prediction, and AHRS ensures consistent heading estimation. Notably, the filter maintains a simplified state representation that tracks only the robot’s two-dimensional position, while the heading angle is provided externally by the AHRS and used as an input during the prediction step. This design avoids estimating the orientation within the filter itself, reducing complexity and computational cost, while still allowing accurate motion prediction by incorporating the robot’s current heading into the odometry model.

The experimental results validate the effectiveness of the proposed system, demonstrating improved accuracy, robustness, and stability while maintaining computational efficiency suitable for deployment on resource-constrained mobile platforms.

This study is also biomimetically inspired, reflecting how living organisms integrate multiple sensory inputs—such as balance, motion, and position—to maintain robust localization in uncertain environments. By fusing UWB, odometry, and AHRS in a lightweight and redundant architecture, the system mimics biological strategies for perception, contributing to the design of energy-efficient and adaptive autonomous agents.

The main contributions of this study are as follows:

- Three-sensor integration: We propose a unified sensor fusion framework that simultaneously incorporates Ultra-Wideband (UWB), wheel odometry, and an Attitude and Heading Reference System (AHRS). This design improves both positional estimation accuracy, particularly in environments with degraded or intermittent signal conditions.
- Real-world validation: The proposed system is implemented on an actual differential-drive mobile robot and evaluated through repeated trajectory tracking trials in realistic indoor environments. These trials include diverse motion dynamics such as frequent turns and variable segment velocities.
- Computational simplicity and robustness assessment: The system employs a standard Kalman filter for real-time sensor fusion, avoiding the complexity and computational cost of approaches like SLAM or factor graph optimization. Furthermore, we introduce Dynamic Time Warping (DTW) [17,18,19] as a trajectory comparison metric to assess localization repeatability across multiple trials—an approach that is more robust to temporal misalignment than traditional pointwise metrics such as RMSE.

The remainder of this paper is organized as follows: Section 2 presents a comprehensive overview of the proposed localization framework, detailing the overall system architecture and sensor configuration. This includes a description of the UWB ranging process and the application of one-dimensional Kalman filters for range denoising, followed by the trilateration method used to compute the robot’s global position. The section further explains the wheel odometry model employed for incremental motion estimation and introduces the Kalman filter-based sensor fusion algorithm that integrates data from the UWB system, wheel encoders, and AHRS to produce a consistent and robust position estimate. Section 3 outlines the trajectory tracking control strategy, which utilizes the fused localization output to ensure accurate and reliable tracking along predefined paths. Section 4 describes the experimental setup and presents the results obtained in real-world environments. Section 5 discusses the evaluation methodology and analysis, including the use of DTW for trajectory comparison and performance interpretation. Finally, Section 6 concludes the paper and outlines potential directions for future research.

## 2. Proposed Localization System

### 2.1. System Overview

This paper proposes a localization system aimed at enhancing the positioning accuracy and reliability of differential-drive mobile robots operating in indoor environments. The system is built upon a modular sensor fusion architecture that integrates three primary sensing modalities: UWB ranging, wheel odometry, and an AHRS. These sensor inputs are fused through a Kalman filter-based algorithm to produce accurate, real-time position estimates.

**Figure 1:**
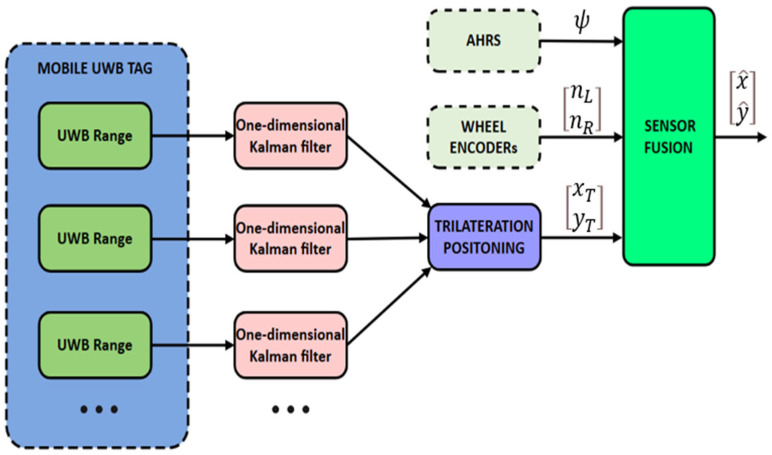
UWB-odometry-AHRS fusion-based localization system.

As illustrated in Figure 1, the system begins by collecting distance measurements from multiple UWB anchors to a mobile UWB tag. For accurate ranging, both the anchors and the tag are ideally mounted at the same height above the ground. When height differences are present, a vertical offset correction is applied by projecting the slant range measurements onto the horizontal plane using the Pythagorean theorem, thereby obtaining equivalent 2D distances. In this setup, UWB anchors are fixed reference nodes with known coordinates, while the UWB tag is a mobile transceiver mounted on the robot. The raw UWB range data, which is often noisy due to multipath effects and interference, is filtered using one-dimensional Kalman filters to reduce uncertainty. The resulting smoothed range measurements are input to a trilateration algorithm, which estimates the 2D position of the mobile tag relative to the anchors.

While the minimum number of anchors required for 2D trilateration is three, the proposed system is designed to support a higher number of anchors if needed. This allows for improved coverage and redundancy in real-world scenarios where signal obstruction or multipath effects may impact individual UWB measurements. When an anchor-to-tag link experiences sudden degradation—such as from occlusion or adverse antenna orientation—the system detects abrupt changes in measured range by calculating the first-order derivative over time. If a rapid deviation is detected, that anchor’s data can be excluded from the trilateration computation to improve robustness. This flexibility enables the system to adaptively reject outlier measurements, supporting more stable and reliable localization in cluttered or dynamic environments.

In parallel, wheel encoders continuously provide odometry data by tracking wheel rotations, and the AHRS supplies heading estimates by fusing accelerometer, gyroscope, and magnetometer inputs. These two sources comprise the motion prediction component of the Kalman-based fusion module.

Within the fusion module, the predicted state from wheel odometry and AHRS is optimally combined with absolute position estimates derived from UWB trilateration. The Kalman filter fuses these inputs to produce robust and accurate real-time localization, effectively mitigating the cumulative drift associated with odometry and the inherent noise in UWB measurements.

Thanks to its modular and sensor-agnostic architecture, the proposed localization framework ensures consistent real-time positioning performance in a variety of indoor settings. It is particularly well-suited for applications such as warehouse automation, service robotics, and the navigation of differential-drive robots in constrained industrial environments.

### 2.2. UWB Ranging and Pre-Processing

Ultra-Wideband (UWB) is a radio communication technology that transmits extremely short pulses across a broad frequency spectrum, typically ranging from 3.1 GHz to 10.6 GHz [4]. Owing to its high temporal resolution, strong resistance to multipath interference, and capacity to deliver centimeter-level positioning accuracy, UWB has emerged as a leading solution for indoor localization applications [4,20]. The core measurement principle employed in UWB localization is the Time of Flight (ToF) method. In this technique, the distance between a UWB transmitter and receiver is estimated by measuring the signal’s propagation time. Specifically, the distance *d* is computed using the following formula:

(1) d=c⋅Δt

where d is the distance, c is the speed of light, and Δt is the measured time delay [21].

UWB signals are particularly well-suited for complex indoor environments, where signal reflections, occlusions, and multipath effects are common. As highlighted by Alarifi et al. [4], when integrated with inertial and odometric data, UWB technology offers robust and accurate positioning under a wide range of environmental conditions. Similarly, Guo et al. [22] demonstrated the successful deployment of UWB in a cooperative localization framework for multi-UAV formation control, underscoring its effectiveness in GPS-denied environments.

Two primary methods are used in UWB localization: Time Difference of Arrival (TDOA) and Two-Way Ranging (TWR). TDOA estimates position based on the time differences in signal arrivals at multiple synchronized receivers. While it can yield high accuracy, it requires strict time synchronization among anchors, making it more suitable for tightly controlled environments [23]. TWR, in contrast, is more practical and widely adopted in mobile and ad hoc systems. It involves the exchange of signals between a pair of devices, and the round-trip time is used to calculate distance. TWR does not require synchronization, which simplifies implementation and makes it ideal for dynamic scenarios [24].

Despite the strengths of UWB, its range measurements are susceptible to transient noise, multipath reflection errors, and non-line-of-sight (NLOS) conditions. To enhance measurement reliability and reduce noise, each raw distance measurement from the UWB module is processed using a one-dimensional Kalman filter (1D-KF). This filtering step helps to stabilize the input data before trilateration. The implementation details of the 1D-KF are provided in Section 2.5.1.

### 2.3. Trilateration-Based Position Estimation

After applying one-dimensional Kalman filtering to the raw UWB range measurements, the resulting smoothed distances are used to estimate the robot’s position through trilateration. Trilateration is a geometric technique that determines the location of a target point by measuring its distances to three or more fixed reference points—referred to as anchors—whose positions are known in a common coordinate frame.

**Figure 2:**
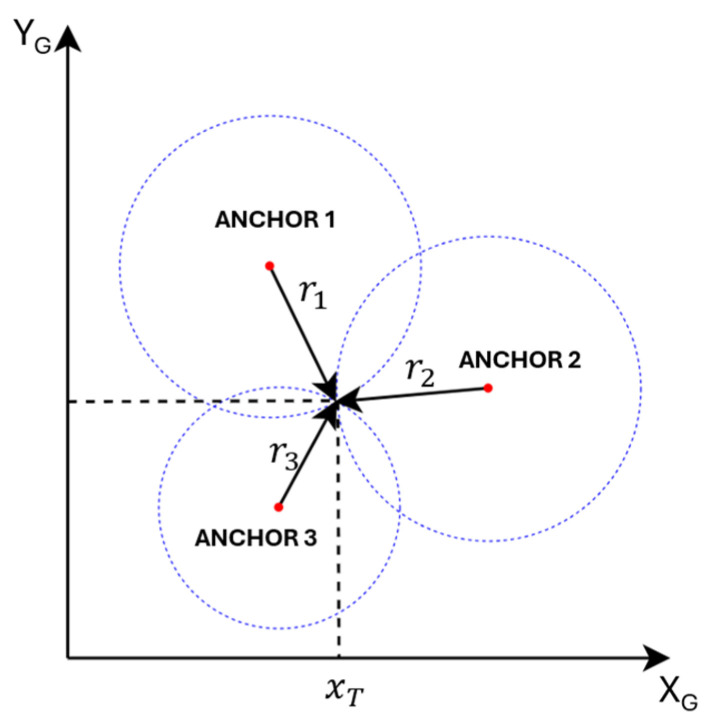
Trilateration system.

In two-dimensional (2D) space, at least three non-collinear anchors are required to unambiguously determine the tag’s coordinates, whereas in three-dimensional (3D) space, four or more non-coplanar anchors are necessary. As illustrated in Figure 2, consider a system where a tag node T has unknown coordinates $\left(x_{T},y_{T}\right)$, and its distances to three anchors $A_{1},A_{2},A_{3}$ located at $\left(x_{A1},y_{A1}\right),\left(x_{A2},y_{A2}\right),\left(x_{A3},y_{A3}\right)$ are given by $r_{1},r_{2},r_{3}$, respectively. However, in practice, the number of anchors can be greater than three and is denoted as n. The square of the Euclidean distance equation for each anchor–tag pair is written as follows:

(2) $$r_{i}^{2}={\left(x_{T}-x_{Ai}\right)}^{2}+{\left(y_{T}-y_{Ai}\right)}^{2},\;i=1,2,\;\ldots ,n$$

Equation (2) represents a set of nonlinear equations with respect to the unknown coordinates ($x_{T}$, $y_{T}$). To linearize the system, the squared distance equation of a selected reference anchor, denoted as $A_{j}$, is subtracted from those of the other anchors. This yields the following linear expression:

(3) $$(x_{Ai}-x_{Aj})x_{T}+(y_{Ai}-y_{Aj})y_{T}=\frac{1}{2}\left[r_{j}^{2}-r_{i}^{2}+x_{Ai}^{2}-x_{Aj}^{2}+y_{Ai}^{2}-y_{Aj}^{2}\right]$$

for any anchor pair i≠j, with i, j ∈{1,…,n}.

When n anchors are available, selecting one as the reference results in a system of n−1 linear equations, which can be compactly written in matrix form as follows:

(4) Ap=b

Here, $p=\left[\begin{array}{c} x_{T}\\ y_{T} \end{array}\right]$ is the vector of unknown tag coordinates, $A\in R^{(n-1)\times 2}$ is the coefficient matrix, and $b\in R^{\left(n-1\right)}$ is a scalar vector.

Specifically, each row of A is constructed as follows:

$$A_{i-1}=\left[\begin{array}{cc} x_{Ai}-x_{Aj} & y_{Ai}-y_{Aj} \end{array}\right]$$

and the corresponding entry of b is given by the following:

$$b_{i-1}=\frac{1}{2}(r_{j}^{2}-r_{i}^{2}+x_{Ai}^{2}-x_{Aj}^{2}+y_{Ai}^{2}-y_{Aj}^{2}),$$

for any anchor pair i≠j, with i, j ∈{1,…,n}.

If exactly three anchors are used (i.e., n=3), the system has a unique solution and the tag position can be directly solved as follows:

(5) $$p=A^{-1}b$$

If more than three anchors are available, the system becomes overdetermined. In this case, the best-fit solution in the least-squares sense is obtained via the Moore–Penrose pseudo-inverse:

(6) $$p={\left(A^{T}A\right)}^{-1}A^{T}b$$

### 2.4. Wheel Odometry

**Figure 3:**
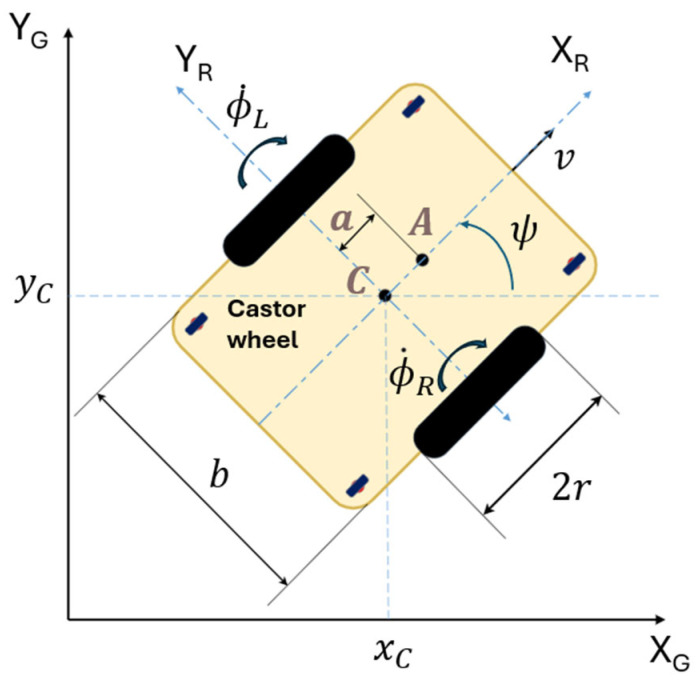
Differential-drive mobile robot.

Wheel odometry is a fundamental dead-reckoning technique for estimating the motion of a differential-drive mobile robot, as illustrated in Figure 3, using encoder measurements from the left and right wheels. Dead-reckoning estimates the current position by integrating incremental motion from a known initial position over time [25].

Consider a scenario where the robot moves over a discrete time interval τ, transitioning from time step k−1 to k. The change in the robot’s pose, expressed as linear displacements (Δx,Δy) and heading variation Δψ, can be described by the following set of equations:

(7) $$\left\{\begin{array}{c} \Delta x=\frac{r}{2}\cdot cos(\psi )\cdot (\Delta \phi_{R}+\Delta \phi_{L})\\ \Delta y=\frac{r}{2}\cdot sin(\psi )\cdot (\Delta \phi_{R}+\Delta \phi_{L})\\ \Delta \psi =\frac{r}{b}\cdot (\Delta \phi_{R}-\Delta \phi_{L}) \end{array}\right.$$

where r is the radius of the wheels, b is the wheelbase (distance between wheels), ψ is the current heading angle of the robot, and $\Delta \phi_{L}$ and $\Delta \phi_{R}$ are the angular displacements of the left and right wheels during the time step.

These angular displacements can be calculated from the encoder tick counts $n_{R}$, $n_{L}\;$, and the encoder resolution $C_{e}$ as follows:

(8) $$\Delta \phi_{R}=\frac{2\pi n_{R}}{C_{e}},\;\Delta \phi_{L}=\frac{2\pi n_{L}}{C_{e}}$$

By defining two constants for compact notation:

(9) $$K_{d}=\frac{\pi r}{C_{e}},\;K_{\psi }=\frac{2\pi r}{bC_{e}}$$

the odometry model can be expressed directly in terms of encoder counts:

(10) $$\left\{\begin{array}{c} \Delta x=K_{d}\cdot cos(\psi )\cdot (n_{R}+n_{L})\\ \Delta y=K_{d}\cdot sin(\psi )\cdot (n_{R}+n_{L})\\ \Delta \psi =K_{\psi }\cdot (n_{R}-n_{L}) \end{array}\right.$$

The robot’s pose in the global frame is then recursively updated as follows:

(11) $$\left\{\begin{array}{c} x_{k}=x_{k-1}+\Delta x\\ y_{k}=y_{k-1}+\Delta y\\ \psi_{k}=\psi_{k-1}+\Delta \psi \end{array}\right.$$

While wheel odometry provides rapid and straightforward motion estimation by integrating encoder data from wheel rotations, it is subject to several inherent limitations that constrain its long-term accuracy and reliability.

Firstly, it requires an initial pose $\left(x_{0},y_{0},\psi_{0}\right)$ to be defined and input manually; otherwise, the robot assumes its starting pose to be zero, which may not align with its actual position. This manual initialization is both time-consuming and prone to errors.

Secondly, odometry suffers from drift over time due to wheel slippage, uneven surfaces, or rapid acceleration and braking. These factors lead to cumulative errors in both position and orientation, which become significant during long-term operation.

As such, while odometry is valuable for short-term motion prediction, it must be augmented by UWB ranging or inertial sensing to ensure long-term localization robustness.

### 2.5. Sensor Fusion Using Kalman Filtering

In this study, sensor fusion techniques are employed to improve the accuracy, consistency, and robustness of localization by combining measurements from multiple heterogeneous sensors. Among the diverse fusion strategies available, Kalman filtering and its variants have emerged as dominant solutions due to their computational efficiency and statistically optimal performance under the assumption of Gaussian noise [26,27,28]. This sections detail the mathematical formulation of the Kalman filter and its specific application to the fusion of odometric and UWB-based measurements within the proposed localization framework.

#### 2.5.1. Kalman Filter

The Kalman filter is one of the most fundamental and widely used algorithms for optimal state estimation in dynamic systems subject to stochastic noise [29]. It provides a recursive solution to the discrete-data linear filtering problem by combining a priori predictions with new measurements to improve accuracy.

The state transition of a linear time-varying system at a discrete time step k is modeled as follows:

(12) $$x_{k}=Fx_{k-1}+{Gu}_{k-1}+\omega_{k-1},\omega_{k-1}∼N\left(0,Q_{k-1}\right)$$

where $x_{k}$ is the system state vector, F is the state transition matrix, G is the control input matrix, $u_{k-1}$ is the control input vector, and $\omega_{k-1}$ represents the process noise, assumed to be zero-mean Gaussian with a covariance matrix $Q_{k-1}$.

Measurements $z_{k}$ are related to the true state $x_{k}$ through the observation model:

(13) $$z_{k}=Hx_{k}+\upsilon_{k},\upsilon_{k}∼N\left(0,R_{k}\right)$$

where H is the measurement matrix and $\upsilon_{k}$ denotes the measurement noise, modeled as zero-mean Gaussian with covariance $R_{k}$**.**

The Kalman filter recursively performs two steps: prediction and update.

**Prediction step:**

The a priori state estimate and its covariance are predicted based on the previous state:

(14) $${\hat{x}}_{k|k-1}=F{\hat{x}}_{k-1}+G_{k}u_{k-1}$$

(15) $$P_{k|k-1}=FP_{k-1}F^{T}+Q_{k}$$

where ${\hat{x}}_{k|k-1}$ is the predicted state and $P_{k|k-1}$ is its associated error covariance.

**Update step:**

When a new measurement becomes available, the innovation and its covariance are computed:

(16) $${\tilde{y}}_{k}=z_{k}-H{\hat{x}}_{k|k-1}$$

(17) $$S_{k}=HP_{k|k-1}H^{T}+R_{k}$$

where ${\tilde{y}}_{k}$ is the innovation matrix, and $S_{k}$ is the innovation covariance.

The Kalman gain is calculated as follows:

(18) $$K_{k}=P_{k|k-1}H^{T}S_{k}^{-1}$$

Finally, the posterior state estimate and its error covariance are updated:

(19) $${\hat{x}}_{k}={\hat{x}}_{k|k-1}+K_{k}{\tilde{y}}_{k}$$

(20) $$P_{k}=\left(I-K_{k}H\right)P_{k|k-1}$$

This recursive process allows the Kalman filter to efficiently fuse prior knowledge and noisy measurements, yielding an optimal state estimate under linear-Gaussian assumptions [26,27,28].

Next, based on the description of the Kalman filter, the one-dimensional Kalman filter introduced in Section 2.2 can be described in terms of the prediction and update steps as follows.

First, in the prediction step:

(21) $${\hat{x}}_{k|k-1}={\hat{x}}_{k-1}$$

(22) $$P_{k|k-1}=P_{k-1}+Q_{k}$$

Second, in the update step:

(23) $$K_{k}=\frac{P_{k|k-1}}{P_{k|k-1}+R_{k}}$$

(24) $$P_{k}=\left(1-K_{k}\right)\cdot P_{k|k-1}$$

#### 2.5.2. Sensor Fusion Using Kalman Filter

To estimate the position of the mobile robot, a Kalman filter is utilized to fuse odometry and measurement data. In this implementation, the key parameters of the Kalman filter, including the state vector, control input, state transition matrix, observation matrix, and noise covariance matrices, are systematically defined as follows. It should be noted that the Kalman filter applied here is not a one-dimensional Kalman filter.

The coordinates of the robot are represented by the state vector $x_{k}$, the estimated state vector ${\hat{x}}_{k}$, and the measurement vector $z_{k}$:

$$x_{k}={\left[\begin{array}{c} x\\ y \end{array}\right]}_{k},\;{\hat{x}}_{k}={\left[\begin{array}{c} \hat{x}\\ \hat{y} \end{array}\right]}_{k},z_{k}={\left[\begin{array}{c} x_{meas}\\ y_{meas} \end{array}\right]}_{k}$$

The prediction of the state vector is based on odometry data, with the control input $u_{k-1}$ and the control matrix **G** defined as follows:

$$u_{k-1}=\left[\begin{array}{c} n_{L}+n_{R}\\ n_{L}+n_{R} \end{array}\right],G=\left[\begin{array}{cc} K_{d}cos\psi & 0\\ 0 & K_{d}sin\psi \end{array}\right]$$

The state transition matrix **F** and the observation matrix **H** are chosen as identity matrices:

$$F={H=I}_{2}$$

where $I_{2}$ is the identity matrix of size 2.

The process noise covariance matrix Q and the measurement noise covariance matrix R are assumed to be constant and are expressed as follows:

$$Q=qI_{2},\;R=\varsigma I_{2}$$

where q and ς are scalar parameters reflecting system and measurement uncertainties, respectively.

## 3. Trajectory Tracking Control

To evaluate the effectiveness of the UWB–Odometry–AHRS fusion-based positioning algorithm in practical autonomous navigation, a trajectory tracking control framework is implemented to verify the stability and reliability of the positioning data. In this context, trajectory tracking is employed as a validation method to assess the accuracy and robustness of the proposed positioning system.

**Figure 4:**
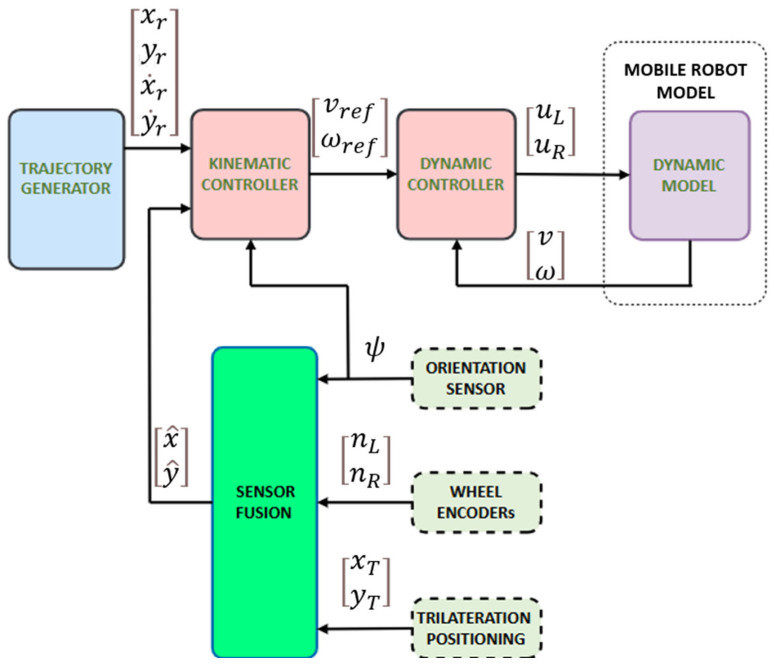
Trajectory tracking control with UWB-based positioning using a Kalman-based sensor fusion technique.

The control architecture is illustrated in Figure 4. A reference trajectory generator provides a sequence of desired values $\left(x_{r},y_{r},{\dot{x}}_{r},{\dot{y}}_{r}\right)$, which are sent to a hierarchical control system consisting of a kinematic controller and a dynamic controller. The kinematic controller produces velocity references $\left(v_{ref},\omega_{ref}\right)$, which are converted into motor commands $\left(u_{L},u_{R}\right)$ by the dynamic controller to actuate the mobile robot. 

Sensor data are processed through the sensor fusion module, which combines encoder readings $\left(n_{L},n_{R}\right)$, orientation ψ from the AHRS sensor, and absolute position measurements $(x_{meas},y_{meas}),$ obtained from UWB trilateration. The output of this module, the estimated position ($\hat{x},\hat{y}$), is then used as feedback for the control system.

The kinematic controller employed in this study is based on the approach proposed by Martins et al. [30]. This controller integrates a nonlinear inverse kinematics model with hyperbolic tangent functions to compensate for tracking errors, ensuring smooth convergence towards the desired trajectory while maintaining system stability. The method effectively addresses the nonlinearities inherent in mobile robot motion and enhances trajectory tracking performance, as demonstrated in previous works by Martins et al. [31], Rossomando et al. [32], Khai & Ryoo [33], and Majid et al. [34].

The control law is formulated as follows:

(25) $$\left[\begin{array}{c} v_{ref}\\ \omega_{ref} \end{array}\right]=\left[\begin{array}{cc} cos\psi & -asin\psi \\ sin\psi & acos\psi \end{array}\right]\left[\begin{array}{c} {\dot{x}}_{r}+l_{x}tanh\left(\frac{k_{x}}{l_{x}}e_{x}\right)\\ {\dot{y}}_{r}+l_{y}tanh\left(\frac{k_{y}}{l_{y}}e_{y}\right) \end{array}\right]$$

where $e_{x}=x_{r}-x$ and $e_{y}=y_{r}-y$ represent the position errors in the $X_{G}$ and $Y_{G}$ axes; ${\dot{x}}_{d},{\dot{y}}_{d}$ denote the desired trajectory velocities; $k_{x}$, $k_{y}$ > 0 are the controller gains; $l_{x}$, $l_{y}$ > 0 are positive saturation constants; ψ is the heading angle, representing the orientation of the robot in the global frame; a is the distance from the center-point ***C*** of the robot to the point ***A***, a point selected on the symmetry axis of the robot’s body, as illustrated in Figure 3.

This transformation enables the conversion of Cartesian velocity references into the robot’s linear and angular velocity commands while considering its orientation and geometric configuration.

The use of the tanh function ensures bounded control output, contributing to smooth control action without introducing excessive actuation effort. This makes the method particularly suitable for mobile robots navigating in indoor environments using UWB localization.

## 4. Experiment

### 4.1. Hardware Setup

**Figure 5:**
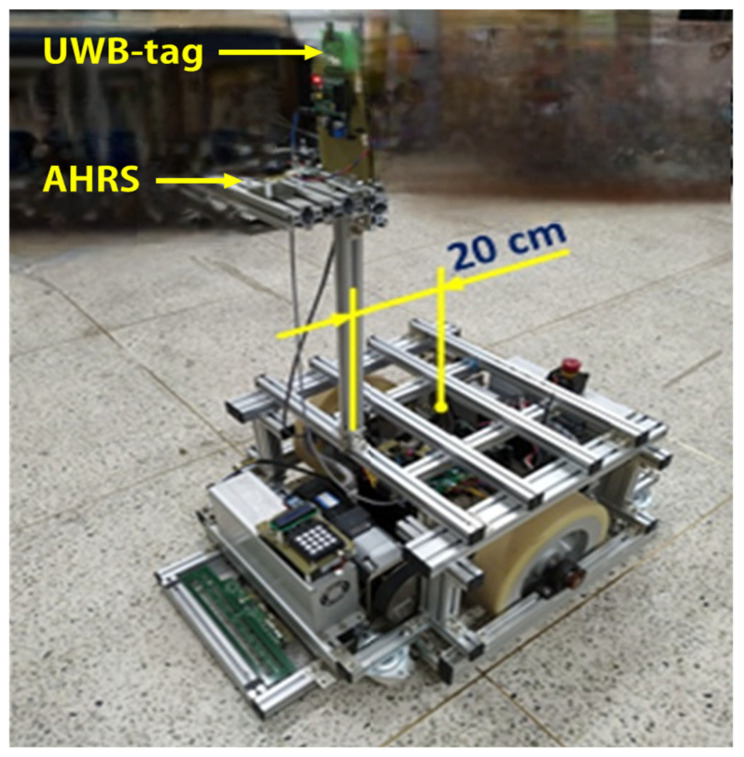
Mobile robot with UWB tag and AHRS.

To evaluate the proposed UWB–odometry–AHRS fusion-based localization system, an experimental platform was constructed using a differential-drive mobile robot, as illustrated in Figure 5. The robot is driven by two TM90-D0431 three-phase, four-pole BLDC motors (400 W, 24 V), and stabilized by a passive caster wheel.

For odometry, integrated hall-effect sensors within the motors generate real-time position updates. Considering a gear ratio of 50:1, each wheel revolution yields 200 discrete pulses, providing sufficient resolution for accurate pose estimation.

Robot orientation is determined using a Sparton GEDC-6 Attitude and Heading Reference System (AHRS), which integrates tri-axial accelerometers, gyroscopes, and magnetometers. The AHRS employs an adaptive filtering algorithm to mitigate magnetic disturbances, ensuring reliable heading estimation in indoor environments. To minimize the impact of vibrations, the AHRS sensor is centrally mounted on the robot chassis.

For absolute position measurements, a UWB positioning system is deployed, in which both the tag and anchor modules consist of a Decawave DWM1000 transceiver integrated with a Waveshare Core103R development board, based on the STM32F103RCT6 ARM Cortex-M3 microcontroller. A single UWB-tag module is mounted on the mobile robot, while three UWB-anchor modules are installed at fixed, known positions within a 5 m × 5 m testing area. Absolute localization is achieved using a UWB positioning system. The UWB tag and anchor modules are equipped with Decawave DWM1000 UWB transceivers, interfaced with Waveshare Core103R development boards based on the STM32F103RCT6 ARM Cortex-M3 microcontroller. A single UWB tag is mounted on the robot, and three UWB anchor nodes are placed at fixed, known locations within a 5 m × 5 m test area.

The UWB system operates on Channel 4 (center frequency: 3993.6 MHz; bandwidth: 900 MHz), with a preamble length of 1024 symbols, a data rate of 6.8 Mbps, and a pulse repetition frequency (PRF) of 64 MHz. These parameters are selected to balance high positioning accuracy with low latency. Communication between the microcontrollers and DWM1000 modules is handled via the SPI protocol.

The overall system was implemented on an embedded hardware platform with constrained computational resources and strict real-time requirements. Each UWB tag module performs onboard trilateration to compute its own position estimate $\left(x_{meas},\;y_{meas}\right)$, which is transmitted to the robot’s main controller via RS232. Empirical testing showed that the position update cycle takes approximately 14 ms.

The main controller is built on an Arduino Mega2560 platform, executing a control loop with a fixed sampling interval of 10 ms. This loop integrates three main tasks: sensor data acquisition from wheel encoders and the AHRS, Kalman filter-based sensor fusion, and the computation of control signals for trajectory tracking. Given that the UWB update rate (~14 ms) is slightly slower than the control loop (10 ms), a buffering mechanism is employed on the main controller to store the most recent UWB data. In each fusion cycle, the latest available position estimate is retrieved and used directly. This asynchronous handling ensures stable sensor fusion performance without introducing excessive delay or inconsistency.

In terms of power consumption, the system is highly efficient and well-suited for embedded applications. The UWB modules (based on the Decawave DWM1000) typically consume under 120–150 mW depending on ranging frequency and duty cycle, while the Arduino Mega2560 operates at less than 0.5 W under typical conditions. This enables the entire localization and control pipeline to run reliably on low-power embedded hardware, without requiring energy-intensive components such as LiDAR, SLAM processing units, or GPUs.

The mobile robot used in this study has a width of 0.558 m, a length of 0.84 m, a wheel diameter of 0.3 m, and an unloaded weight of 125 kg. It supports a maximum payload of 400 kg and can reach a top linear speed of 0.936 m/s. All the experiments were conducted in a flat, obstacle-free indoor laboratory environment. A summary of the key kinematic and physical parameters of the mobile robot is presented in Table 1.

To prevent signal obstruction caused by obstacles moving around the mobile robot, the UWB tag module is mounted the top of the pillar on the mobile robot. This pillar is positioned along the longitudinal symmetry plane of the mobile robot and offset by a distance of L=20 cm from the center of the wheel axis. In the experimental setup, the height of the DWM1000 module above the ground is approximately 110 cm. Given the coordinates of the UWB tag $\left(x_{T},y_{T}\right),$ the offset L and the heading angle ψ, the position of the mobile robot $\left(x_{meas},y_{meas}\right)$ can be defined as follows:

(26) $$\left\{\begin{array}{c} x_{meas}=x_{T}-Lcos\psi \\ y_{meas}=y_{T}-Lsin\psi \end{array}\right.$$

### 4.2. Experimental Results

During the experiments, three UWB anchor modules were placed at fixed, known locations within the testing area: Anchor 1 at (0.00 m, 0.00 m), Anchor 2 at (5.52 m, 0.89 m), and Anchor 3 at (1.84 m, 3.455 m). These anchors formed an irregular triangle to ensure sufficient geometric diversity, thereby enhancing trilateration accuracy.

In the trajectory tracking controller, the distance a was set to 0.2 m. The parameters of the kinematic controller were configured as *k_x_* = 0.4, *l_x_* = 1, *k_y_* = 0.4, and *l_y_* = 1. For the proposed Kalman-based sensor fusion positioning system, the covariance matrix parameters were set to $q=0.0012\cdot 10^{-3}$ and ς=0.046. These parameters were selected through empirical tuning based on observed system behavior and performance during testing.

To evaluate the performance and generalization capability of the proposed localization system, two experimental scenarios with different levels of trajectory complexity were conducted. In both cases, the mobile robot was required to follow a predefined multi-segment path using the same control and sensor fusion framework. The first scenario employed a simple rectangular loop, while the second involved a more complex trajectory with additional turning segments. Details of each scenario are provided below.

#### 4.2.1. First Experimental Scenario

In the first experimental scenario, the mobile robot was tasked with following a segmented trajectory consisting of four sequential path segments (Segment 1 to Segment 4). Each segment was defined by a start and end point, as listed in Table 2. The mobile robot was required to move through these segments in the specified order, completing one segment before proceeding to the next.

To generate the desired trajectory for the mobile robot, a reference velocity $v_{r}$ was introduced as an intermediate variable to compute the reference trajectory states $\left(x_{r},y_{r},{\dot{x}}_{r},{\dot{y}}_{r}\right)$. This reference velocity was not used for direct control, but rather served to continuously generate the desired position and velocity profiles. At the start of each trajectory segment, $v_{r}$ was initialized and evolved following a linear ramp profile during the acceleration phase, and subsequently maintained at a constant value. Specifically, $v_{r}$ was defined as follows:

$$\mathrm{if}\;t<1\;s,\;\mathrm{then}\;v_{r}=\frac{v_{r}^{max}}{T}\cdot t,\;$$

$$\mathrm{else}\;v_{r}=v_{r}^{max}$$

where $v_{r}^{max}=0.157\;m/s$ denotes the maximum reference velocity, T=5 s, and t=0 s represents the start time of each segment.

The mobile robot was instructed to follow the predefined trajectory repeatedly over 14 laps. This repetition enabled the evaluation of trajectory tracking consistency and stability using the proposed sensor fusion method, and also facilitated comparison with a baseline UWB-only approach.

**Figure 6:**
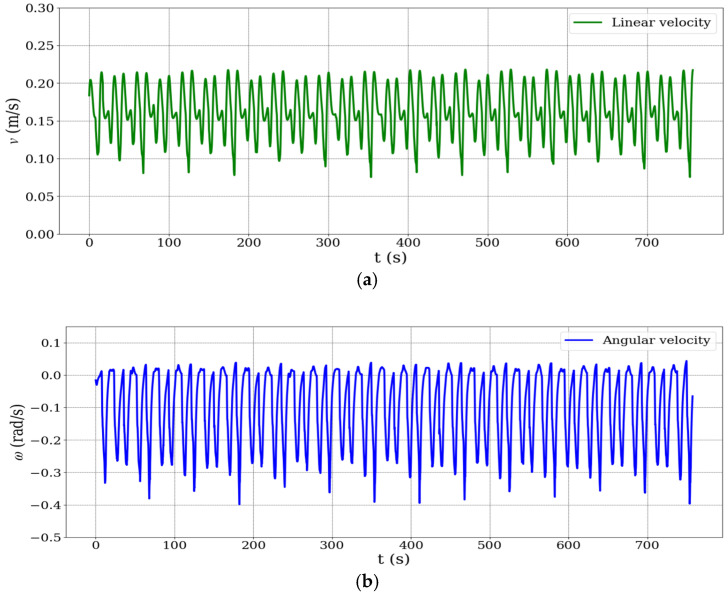
Time series plot of the mobile robot’s motion profiles: (**a**) linear velocity v in meters per second; (**b**) angular velocity ω in radians per second.

Figure 6 presents the time series plots of the mobile robot’s (a) linear velocity and (b) angular velocity. Although no external disturbances were applied, the robot’s velocity profile shows natural fluctuations due to trajectory execution dynamics. The robot was operated at low speeds for control stability, but its relatively large mass required significant deceleration during cornering and re-acceleration along straight paths. These repeated changes in speed imposed internal dynamic challenges—such as wheel slip and inertia effects—which could degrade odometry accuracy. As such, the non-uniform velocity profile implicitly served as a realistic test of the robustness of the proposed sensor fusion framework under variable motion conditions.

As illustrated in Figure 6b, the angular velocity of the mobile robot exhibits periodic fluctuations reaching approximately −0.4 rad/s. These variations are primarily caused by the robot’s rotational maneuvers at trajectory segment transitions, particularly during 90° turns along the rectangular path. Such behavior is characteristic of differential-drive mobile robots, which rely on differential wheel speeds to execute turning actions.

To manage these dynamics, the nonlinear kinematic controller implemented in this study uses bounded hyperbolic tangent functions to limit control outputs during sharp heading changes, thereby preventing actuator saturation and ensuring motion smoothness. Despite the presence of significant angular velocity variations, the Kalman filter-based sensor fusion algorithm remained stable throughout all the experiments, with no signs of divergence or instability in the estimated position. This demonstrates the robustness of the proposed localization system under high angular dynamics and abrupt heading transitions.

Throughout the trials, both the raw UWB trilateration position measurements $\left(x_{meas},\;y_{meas}\right)$ and the fused position estimates ($\hat{x},\hat{y}$) were recorded in real time for subsequent localization performance analysis.

**Figure 7:**
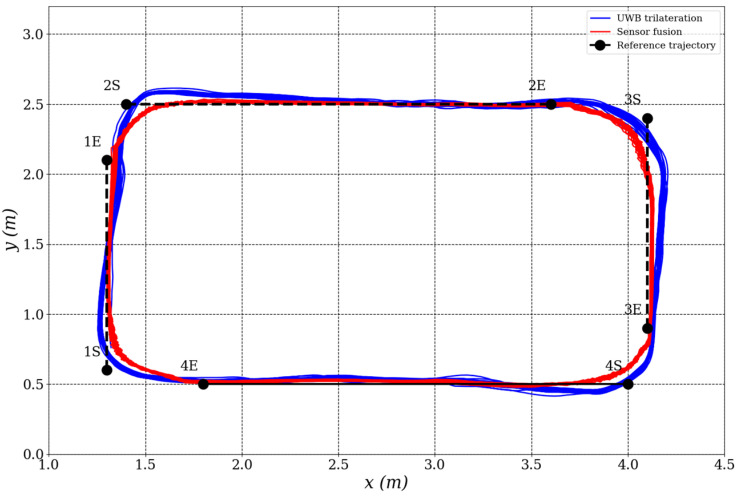
Trajectory tracking performance of the mobile robot in the first experimental scenario.

Figure 7 illustrates the mobile robot’s trajectory tracking performance. The trajectory derived from sensor fusion (red solid line) aligns more closely with the reference path (blue dashed line) compared to the raw UWB trilateration trajectory (green dashed line), which exhibits noticeable fluctuations and deviations—especially at segment transitions and turning points. These results underscore the benefits of sensor fusion in suppressing measurement noise and improving localization reliability.

**Figure 8:**
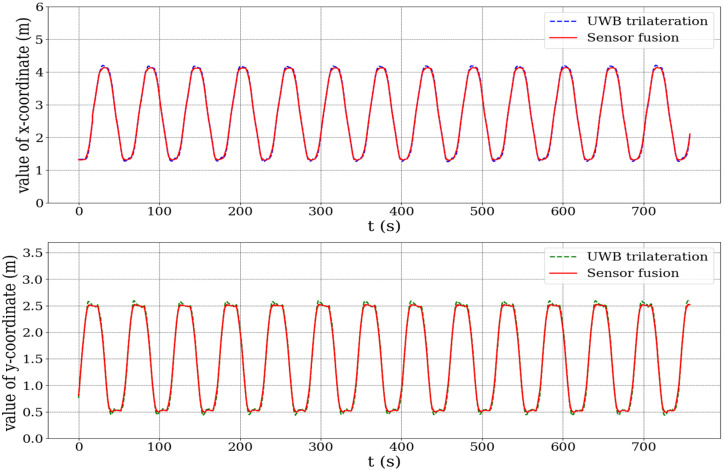
Time series plot of *x* and *y* positions for UWB trailateration and sensor fusion positioning.

To further assess the localization system’s dynamic behavior, Figure 8 presents a time series plot of the *x* and *y* positions for both localization methods. The sensor fusion results display significantly smoother signals, characterized by reduced noise and consistent trajectory tracking. In contrast, the UWB trilateration data show more oscillatory behavior, likely due to environmental noise and multipath signal reflections commonly observed in indoor localization scenarios.

#### 4.2.2. Second Experimental Scenario

In the second experimental scenario, the mobile robot was instructed to follow a more complex trajectory composed of eight sequential path segments (Segment 1 to Segment 8). This extended trajectory was designed to introduce sharper turns and more varied motion patterns in order to evaluate the adaptability and robustness of the proposed localization system under increased trajectory complexity. Each segment was defined by a start and end point, as detailed in Table 3.

**Figure 9:**
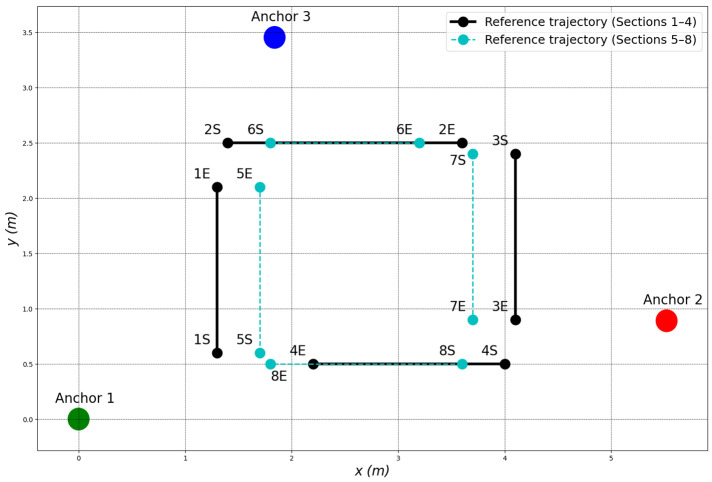
Reference trajectory for the second experimental scenario.

The corresponding reference trajectory, which served as the desired path for the robot to follow, is illustrated in Figure 9.

The reference velocity profile in the second scenario followed the same configuration as in the first, with a linear ramp during acceleration and a constant cruising speed thereafter. The robot completed six full laps along the 8-segment path to evaluate the generalization ability of the proposed fusion method under increased trajectory complexity.

**Figure 10:**
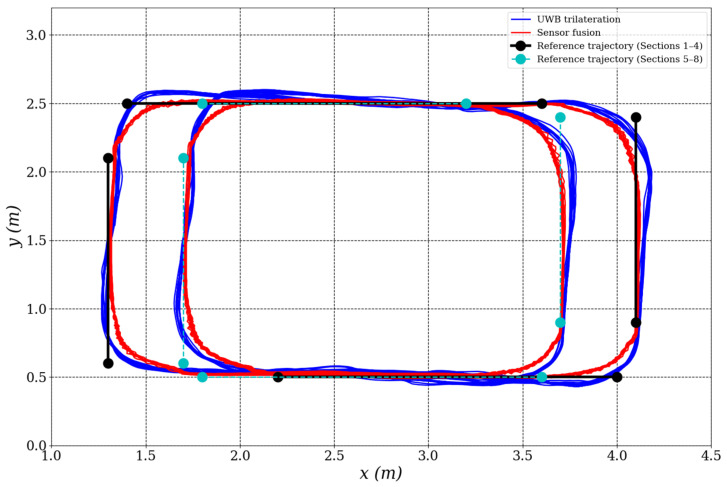
Trajectory tracking performance of the mobile robot in the second experimental scenario.

The time series plots of position (*x*,*y*) and velocity signals (linear and angular) observed in this scenario were qualitatively similar to those in the first scenario, and thus are not repeated here. Instead, the evaluation focuses on the spatial trajectory results. As shown in Figure 10, the fused position estimates ($\hat{x},\hat{y}$) exhibit smoother and more consistent paths than raw UWB trilateration position measurements $\left(x_{meas},\;y_{meas}\right)$, further demonstrating the robustness of the proposed localization framework.

## 5. Discussion

### 5.1. Experimental Methodology

To rigorously assess the similarity between the executed trajectories and the reference paths across multiple trials, Dynamic Time Warping (DTW) was employed as the principal comparison metric. DTW offers a distinct advantage in this context because it enables flexible, nonlinear temporal alignment between trajectory sequences, thereby compensating for variations in execution speed, control latency, and minor temporal irregularities that commonly arise in practical robotic systems.

Unlike conventional pointwise error metrics, such as Root Mean Square Error (RMSE), which require perfectly time-synchronized data and can yield misleading similarity assessments when trajectories exhibit local temporal shifts, DTW computes an optimal warping path that minimizes cumulative spatial deviations irrespective of temporal correspondence. Prior research has demonstrated that pointwise metrics tend to overestimate dissimilarity when sequences experience speed variations or asynchronous sampling, leading to an underestimation of true spatial similarity [35]. In contrast, DTW has been widely validated as a robust approach in trajectory analysis tasks where repeated motions are subject to non-uniform temporal distortions [17,18,19].

These properties make DTW particularly well-suited for evaluating mobile robot navigation accuracy under real-world conditions, where environmental uncertainties and system response delays often preclude strict temporal alignment between repeated trajectories.

The DTW distance between two trajectories

$$P=\{p_{1},p_{2},\ldots ,p_{N}\},\;\;Q=\{q_{1},q_{2},\ldots ,q_{M}\}$$

where $p_{k}=[x_{k},y_{k}]$ and $q_{l}=[{x^{\prime }}_{l},{y^{\prime }}_{l}]$ is computed as follows:

(27) $$DTW(P,Q)={min}_{\omega \in \Omega }\sum_{(k,l)\in \omega }\|p_{k}-q_{l}{\|}_{2}$$

Here, Ω denotes the set of all the admissible warping paths, and $\|\cdot {\|}_{2}$ represents the Euclidean distance between spatial points.

In this study, pairwise DTW distances were computed between multiple repetitions of the mobile robot’s trajectories under two localization configurations: (1) UWB trilateration and (2) the proposed fusion-based method. The resulting distance matrices were analyzed to assess trajectory consistency across repetitions. Lower intra-class DTW distances indicate higher repeatability and localization precision.

This experimental methodology provides a rigorous and temporally flexible comparative framework, clearly demonstrating the benefits of the fusion-based localization strategy relative to the baseline UWB method.

### 5.2. Analysis

To quantitatively evaluate the consistency of trajectory execution across different localization strategies, Dynamic Time Warping (DTW) distances were computed between repeated trajectories in two experimental scenarios. For each method, a pairwise DTW distance matrix was generated based on the two-dimensional position data.

**Figure 11:**
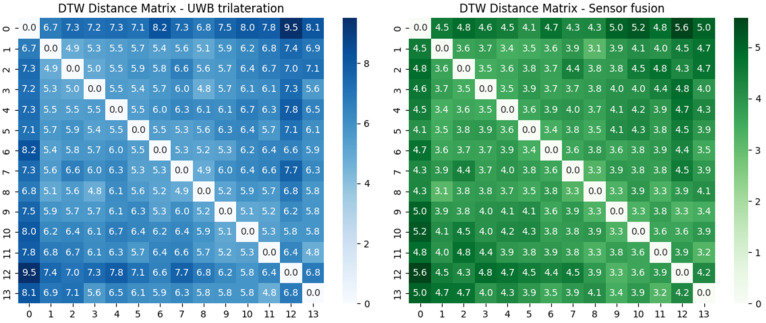
Dynamic Time Warping (DTW) distance matrices for UWB trilateration (**left**) and sensor fusion (**right**) in the first scenario.

Figure 11 illustrates the DTW distance matrices for the first scenario. The left matrix shows DTW distances calculated from raw UWB trilateration-based positions ($x_{meas},\;y_{meas}$), while the right matrix represents distances obtained from the proposed sensor fusion-based localization ($\hat{x},\hat{y}$). Each matrix element quantifies the spatial–temporal similarity between two trajectories using DTW.

Scenario 1 results:

- Mean DTW distance—UWB trilateration: 6.22;
- Mean DTW distance—Sensor fusion: 4.01.

These results demonstrate that the proposed sensor fusion method yields significantly more consistent trajectory execution compared to UWB-only localization.

**Figure 12:**
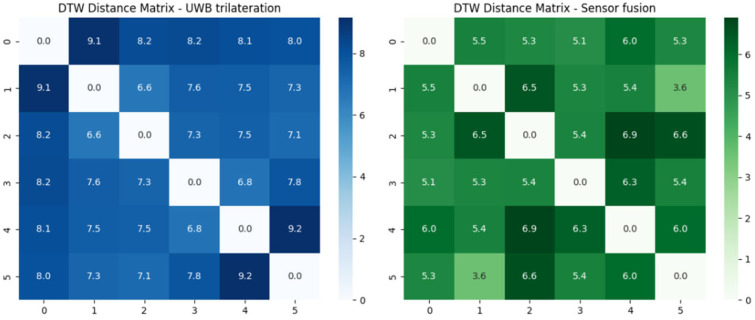
Dynamic Time Warping (DTW) distance matrices for UWB trilateration (**left**) and sensor fusion (**right**) in the second scenario.

Figure 12 presents the DTW distance matrices for the second scenario, following the same evaluation procedure. Once again, the left matrix corresponds to UWB trilateration, and the right to sensor fusion-based localization.

Scenario 2 results:

- Mean DTW distance—UWB trilateration: 7.74;
- Mean DTW distance—Sensor fusion: 5.65.

Across both scenarios, the sensor fusion approach consistently results in lower DTW distances, confirming its effectiveness in improving trajectory repeatability and reducing localization noise.

It is worth noting that the DTW distances observed in Scenario 2 are generally higher than those in Scenario 1 for both localization methods. This can be attributed to several factors related to the trajectory structure and sensor limitations. First, the trajectory in Scenario 2 includes more segments and sharper turns, resulting in more complex and dynamic motion patterns. These variations increase the difficulty of achieving consistent spatial–temporal alignment across multiple repetitions, naturally leading to higher DTW values. Moreover, in sharp turns, the sensor fusion approach may suffer from increased noise, particularly due to wheel encoder inaccuracies. During cornering, wheel slippage can occur, which degrades the accuracy of odometry measurements. Since the fusion algorithm relies partially on these inputs, such disturbances can propagate into the estimated positions, especially in curved segments. This effect is less prominent in straight-line motion, which dominates Scenario 1. Therefore, the increase in DTW distances in Scenario 2 likely reflects a combination of trajectory complexity and sensor-related noise under dynamic conditions, reinforcing the importance of robust fusion strategies in environments with frequent directional changes.

These results demonstrate that the trajectories generated by the sensor fusion method are more consistent across laps, as reflected by the lower mean DTW distance. This improvement can be attributed to the fusion method’s ability to mitigate the effects of multipath interference, transient noise, and non-line-of-sight conditions that affect raw UWB measurements.

Additionally, the distance matrix from the sensor fusion approach exhibits reduced variance and more uniform pairwise distances, further supporting its enhanced stability and robustness. In contrast, the UWB-only matrix shows greater fluctuation between trials, highlighting its susceptibility to environmental variability.

In summary, the experimental findings demonstrate that the proposed sensor fusion-based localization system significantly enhances the precision, robustness, and repeatability of a mobile robot’s trajectory tracking in structured indoor environments. By reducing localization noise and improving trajectory fidelity, the fusion method offers a more reliable solution for autonomous navigation tasks.

## 6. Conclusions

This study proposed and experimentally validated a position estimation approach for a mobile robot based on Kalman-filtered fusion of UWB trilateration, wheel odometry, and AHRS data. A hierarchical control framework was implemented, incorporating a nonlinear kinematic controller with hyperbolic tangent compensation to ensure smooth and stable trajectory tracking.

The proposed localization strategy integrated raw UWB trilateration measurements with encoder and inertial orientation data from the AHRS through a Kalman filter, enabling more accurate and robust position estimation. The experimental results, obtained through repeated trajectory tracking along a closed-loop path, demonstrated that the fusion-based method significantly outperformed UWB trilateration alone, yielding improved positioning accuracy, reduced noise, and enhanced trajectory consistency. The trajectories generated by the sensor fusion method are more consistent across laps, as reflected by the lower mean DTW distance.

The configuration of the Kalman filter was achieved through a streamlined calibration process, requiring only a limited number of test iterations to achieve stable and accurate performance. The parameter adjustment was consistent across runs and did not demand extensive effort or expertise. As a result, the system remains easy to deploy and adapt, with minimal tuning effort even when transferred to different robot platforms or indoor environments.

Overall, the proposed method offers a practical and effective solution for improving mobile robot localization in indoor environments, contributing to more robust and precise autonomous navigation. Future research will explore the integration of additional sensing modalities and the development of adaptive fusion algorithms to further enhance performance under dynamic and uncertain conditions. Although the floor surface used in our laboratory experiments was generally flat, minor variations in surface texture and roughness were naturally present across different areas of the testing environment. However, other real-world disturbance factors such as slippery surfaces or reduced traction—common in industrial settings—were not explicitly considered and will be investigated in future work to further evaluate the robustness of the fusion framework under such conditions.

## Figures and Tables

**Table 1:** Kinematic and physical parameters of the mobile robot.

Parameter	Value	Description
Wheel radius (r)	0.15 m	Radius of each drive wheel
Wheelbase (b)	0.558 m	Distance between the centers of the left and right wheels
Wheel diameter	0.3 m	Measured as 2 × wheel radius
Robot length	0.84 m	Total longitudinal length of the chassis
Robot width	0.558 m	Same as the wheelbase due to axle-mounted wheels
Encoder resolution ($C_{e}$)	200 ticks/rev	Pulses per revolution (after gear ratio 50:1)
Maximum linear speed	0.936 m/s	Peak linear velocity of the robot
Unloaded weight	125 kg	Without payload
Maximum payload	400 kg	Designed load capacity

**Table 2:** Start and end points of each trajectory segment in the first experimental scenario.

Segment	Start Point (S) (m)	End Point (E) (m)
Segment 1	(1.3, 0.6)	(1.3, 2.1)
Segment 2	(1.4, 2.5)	(3.6, 2.5)
Segment 3	(4.1, 2.4)	(4.1, 0.9)
Segment 4	(4.0, 0.5)	(1.8, 0.5)

**Table 3:** Start and end points of each trajectory segment in the second experimental scenario.

Segment	Start Point (S) (m)	End Point (E) (m)
Segment 1	(1.3, 0.6)	(1.3, 2.1)
Segment 2	(1.4, 2.5)	(3.6, 2.5)
Segment 3	(4.1, 2.4)	(4.1, 0.9)
Segment 4	(4.0, 0.5)	(1.8, 0.5)
Segment 5	(1.7, 0.6)	(1.7, 2.1)
Segment 6	(1.8, 2.5)	(3.2, 2.5)
Segment 7	(3.7, 2.4)	(3.7, 0.9)
Segment 8	(3.6, 0.5)	(1.8, 0.5)

## Data Availability

The raw data supporting the conclusions of this article will be made available by the authors on request.
